# Soil Parameter Mapping and Ad Hoc Power Analysis to Increase Blocking Efficiency Prior to Establishing a Long-Term Field Experiment

**DOI:** 10.1155/2015/205392

**Published:** 2015-07-13

**Authors:** Doug Collins, Chris Benedict, Andy Bary, Craig Cogger

**Affiliations:** ^1^Center for Sustaining Agriculture and Natural Resources, Washington State University, 2606 W Pioneer, Puyallup, WA 98371, USA; ^2^Washington State University Extension, 1000 N Forest Street, Suite 201, Bellingham, WA 98225, USA; ^3^Washington State University Crop and Soil Sciences, 2606 W Pioneer, Puyallup, WA 98371, USA

## Abstract

The spatial heterogeneity of soil and weed populations poses a challenge to researchers. Unlike aboveground variability, below-ground variability is more difficult to discern without a strategic soil sampling pattern. While blocking is commonly used to control environmental variation, this strategy is rarely informed by data about current soil conditions. Fifty georeferenced sites were located in a 0.65 ha area prior to establishing a long-term field experiment. Soil organic matter (OM) and weed seed bank populations were analyzed at each site and the spatial structure was modeled with semivariograms and interpolated with kriging to map the surface. These maps were used to formulate three strategic blocking patterns and the efficiency of each pattern was compared to a completely randomized design and a west to east model not informed by soil variability. Compared to OM, weeds were more variable across the landscape and had a shorter range of autocorrelation, and models to increase blocking efficiency resulted in less increase in power. Weeds and OM were not correlated, so no model examined improved power equally for both parameters. Compared to the west to east blocking pattern, the final blocking pattern chosen resulted in a 7-fold increase in power for OM and a 36% increase in power for weeds.

## 1. Introduction

The ability for a researcher to elucidate differences and interactions between imposed treatments is affected by variation between experimental units. While homogeneous experimental units increase the precision of the comparison, results from a narrow population of units can be difficult to extrapolate to other conditions [1]. Agricultural field studies that use experimental units large enough to accommodate typical equipment and a range of field conditions are more likely to produce transferable knowledge but also more likely to encounter heterogeneous experimental units.

Blocking, which has its origins in agricultural research, is a common method to control environmental variation. In a typical complete block design, contiguous plots are placed in a group and the suite of treatments is assigned randomly to the units. This is repeated for more sets of contiguous plots, with each group of treatments representing an experimental replication [1]. Blocking is valuable in field studies when there is variability in a parameter that affects response variables of interest across the landscape and this variability is known and accounted for in homogeneous blocks. To most effectively decrease error variance through blocking, blocks should be formulated so that variation of critical parameters is minimized within blocks [2].

The power of a statistical test is the probability of correctly rejecting the null hypothesis. Accounting for error variance caused by a nuisance factor increases statistical power and the ability to detect differences between treatments. If there is an actual difference between treatments but the hypothesis test does not reject the null hypothesis when it is false, then a type II error has been made. Erroneous conclusions from data where no difference in treatments was detected can be costly and prevent adoption of improved practices [3]. Incorporating a priori understanding of the variability of relevant soil parameters into experimental and sampling designs can increase power and decrease the number of samples required to detect treatment differences [2, 4].

For some experimental units, a priori bias is clear and easily accounted for in block designs. In an animal feeding study, for example, animals (units) can be grouped by size, since one would expect that initial size could impact not only final size but also rate of gain. In heterogeneous landscapes, where the target response variable is soil microorganisms, plants may serve as indicators of below-ground hot spots because of the relationship between primary producers and primary decomposers [4]. However, the precise spatial scale of this relationship is not consistent across all groups of microorganisms [4]. In annual cropping systems, where plant cover is absent or homogeneous, some type of prior investigation and mapping is required to assess spatial variability in soil parameters and proceed with blocking. Power analysis can be incorporated into the investigation to objectively evaluate different blocking schemes.

Inherent soil properties, such as soil texture, can have a significant effect on many parameters typically assessed in cropping systems. Increasing clay content was associated with increased soil aggregation [5, 6], decreased microbial activity [7], decreases in specific pathogenic nematode populations [8], and larger weed populations [9]. Inherent soil properties can also interact with treatment effects; Hao and Kravchenko [10] found that the potential for soil carbon accumulation in reduced tillage treatments varied with clay + silt content.

In this paper we detail the process of gathering preliminary data for two response variables from a field site before assigning treatments to blocks. The experiment evaluates reduced tillage in organic agriculture and was planned to continue for a minimum of 6 years (currently in year 4). The primary goals of the experiment are to evaluate changes in soil quality and weed pressure in the different systems.

Organic farmers typically employ combinations of soil inversion (with a moldboard plough), chisel ploughing, disking, spading, rototilling, and finishing tools to terminate cover crops, incorporate crop residue, control weeds, and prepare the seed bed for planting. Postplant cultivation for weed management is also standard practice to uproot or bury emerged weeds. Conservation or reduced tillage systems avoid soil inversion and reduce the depth of tillage from a typical 25–30 cm to 5–20 cm and also leave at least 30% of the soil surface covered with crop residue after seeding [10, 11]. Zero- or no-till practices are an extreme form of reduced tillage that limit soil disturbance to only what is necessary to place a seed, such as disk openers preceded by narrow cutting coulters [12]. The experiment has four replications, six cropping systems (main plots) and 3 cash crops (subplots) which are rotated annually within the cropping system.

Our primary goals in the long-term experiment are to detect treatment differences in soil quality and weed pressure. The objective of the a priori analysis was to use preliminary information about the spatial variability of these soil properties within our site to determine the most efficient blocking pattern, thereby reducing overall experimental variance and increasing power.

## 2. Methods

### 2.1. Site Description

**Figure 1:**
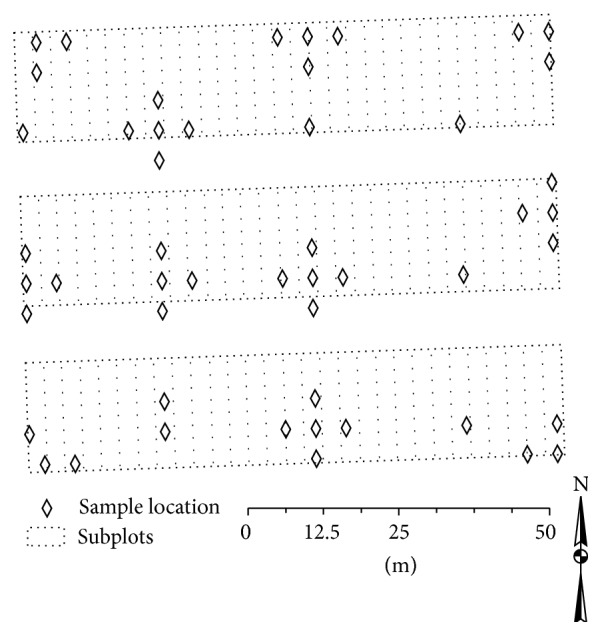
Soil organic matter and weed seed bank samples were taken from sample locations (diamonds). Rectangles represent potential experimental subplots, Puyallup, WA 2011.

The soil is classified as Puyallup fine sandy loam (coarse-loamy over sandy or sandy-skeletal, isotic over mixed, and mesic Vitrandic Haploxerolls). The experimental design for the long-term reduced tillage experiment was a split-plot including 6 main treatments (tillage type), 3 subtreatments (cash crop), and 4 replications. The area available for the experiment was 88.4 × 73.2 meters (0.65 hectares). Due to the size and shape of the area, desired size for subplots and alley ways, and irrigation considerations, plots were oriented in a north-south direction. The entire area included 87 subplots (Figure 1) but only 72 were to be used for the final experiment. Subplots were 3.0 × 18.3 meters, main plots were 9.1 × 18.3 meters, and alleys were 9.1 meters wide.

### 2.2. Soil and Weed Sampling

The corners for the sample area were identified with a global positioning unit (GEO Explorer 6000, Trimble Navigation Limited, Sunnyvale, CA). Using ArcMap (ArcGIS, ESRI Redlands, CA) we located fifty sites across the experimental area with a minimum distance between sample locations of 5 m (Figure 1). Sample site locations were loaded onto the GPS unit and soil was collected from the georeferenced sites on December 11, 2011, by taking 3 cores (44 mm diameter) to a depth of 150 mm within a 0.3 × 0.3 m area at each site. This depth was chosen to be within the traditional tillage zone. This zone typically has the largest organic matter content [13] and most uniform weed seed bank [14] and is where subsequent treatment effects are expected to be the most pronounced. Samples were mixed well and split for organic matter and weed seed bank density evaluation.

Organic matter was evaluated via loss on ignition; soils were air-dried, processed in a soil grinder, and sieved to 2 mm before being combusted in a muffle furnace at 360 C for 2 hrs [15]. Two subsamples were combusted and averaged before further analysis.

Weed seed bank density was determined through a direct germination method as described by Gross [16]. One kilogram of air-dried soil was mixed with 500 g soilless media and placed in flats in a greenhouse (day:night temperature: 21.1:15.6 C and day:night duration 14:10 hrs) and watered daily. Germinated weeds were counted by species then stirred to invigorate germination. Counts continued until no germination occurred for 2 weeks and then the soil was placed into a freezer for 1 month and the described process continued until no germination occurred for 1 month. Weed seed bank data was tallied for total weeds (TTL), broadleaf weeds (BRDLF), total grasses (GRASS), mustard species (MUST),* Lamium amplexicaule* (LAMAM), and* Stellaria media* (STEME). These categories were chosen based on dominant species present.

### 2.3. Exploratory Data Analysis

Summary statistics were calculated for each parameter and a correlation matrix was developed from all weed and organic matter data. Site-specific values for organic matter and total weeds were displayed with post maps and bubble plots to examine for erratic data [17]. Based on a posting of the data one soil organic matter point was deemed to be erratic and further explored. Plotting variograms with and without the point demonstrated a more structured variogram without the point, so it was removed from the carbon analysis before computing the final variogram. No points were removed from the total weed analysis.

### 2.4. Analysis of Spatial Variability and Mapping

Semivariograms were calculated for organic matter and total weed density with the variogram function in the* gstat* package in R [17, 18]. After fitting the appropriate model, maps of organic matter and total weed density were predicted onto a 1 m grid using* predict.gstat* and exported as ASCII files and then imported into ArcMap. A polygon file showing subplots was created in ArcMap. Mean organic matter and total weed densities were calculated on the subplot level with the* zonal statistics to table* tool in ArcMap.

Subplot-level data were ranked by value and visually inspected. Different blocking schemes were created by inspection of the data to try and independently maximize homogeneity for organic matter and total weeds within a block. With the space available there was room for 87 subplots, though only 72 were necessary for the experiment. Therefore, 15 subplots were not used in each design. Four different blocking designs were created: (1) stacked replications moving from west to east, (2) organic matter optimization, (3) weed optimization, version 1, and (4) weed optimization version 2.

**Figure 2:**
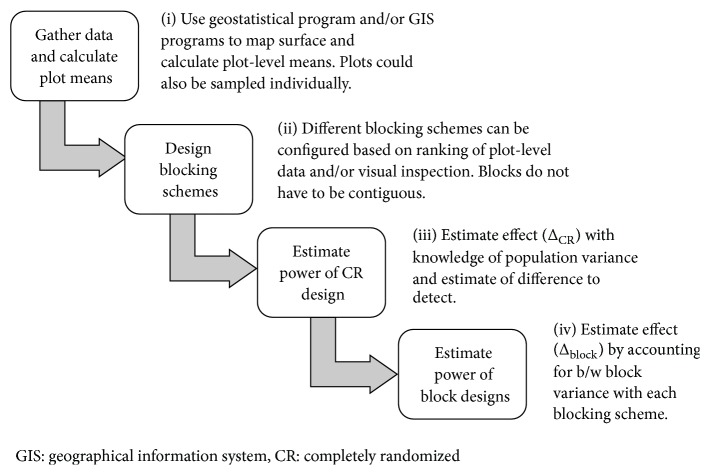
Diagram of process used to gather preliminary data, portray spatial variability in subplots, and analyze power of different blocking patterns.

The sub-plot-level data generated in ArcMap were coded for each blocking scenario (e.g., block number 1–4 or 0 if excluded) and exported for power analyses. The process used for gathering preliminary data and modeling the power of specific block designs is diagramed in Figure 2.

### 2.5. Ad Hoc Power Analysis

Statistical power is affected by 4 variables: significance level (*α*), number of replications (*n*), the effect size (Δ or *f*), and the number of groups or treatments (*k*). The effect size is an index of how big or small of a difference between treatments that one wants to be able to detect. It is the ratio of standard deviation of population means to the standard deviation of populations [19]:

(1) Δ=σuσ,σμ=∑i=1kμi−μ2k,

where *σ*_*μ*_ = variation between overall mean and expected mean and *σ* = error variation.

The variation between the overall mean and the expected mean (*σ*_*μ*_) was calculated for organic matter and total weeds by using the overall mean of each response variable from the preliminary. For total weeds, we assumed that a 10% difference in population means should be detected. In other words, we calculated the power of the statistical test to accurately reject the null hypothesis if there was a difference between two population means of at least 10%. For organic matter, we assumed a 5% difference in population means should be detected.

Power analysis was done with the* pwr* package [20]. A subset of the full dataset was taken for each blocking scheme to exclude subplots not included in that model. Remaining subplots were then collapsed into main plots (*n* = 24) by combining and averaging 3 contiguous subplots. Six treatments were randomly assigned to the main plots as both a completely randomized (CR) design with 4 replications and also as a randomized complete block (RCB) with 4 blocks. The Δ_CR_ value is used to calculate both CR power and RCB power, so CR power was calculated first. An *α* of 0.05, an *n* of 4, and a *k* of 6 were used for all tests. The standard deviation within the population means, *σ*, was calculated as the treatment variance plus the error variance following analysis of variance. The blocked effect size (Δ_block_) was calculated for each randomized block design and used in the power analysis. Blocked effect size is determined as

(2) $$\begin{array}{c} \Delta_{\text{block}}=\frac{\Delta_{\text{CR}}}{\sqrt{1-{\text{PV}}_{b}}}, \end{array}$$

where Δ_block_ = effect size of RCB design, Δ_CR_ = effect size of analogous completely randomized design, PV_block_ = *σ*_block_^2^/*σ* ^2^ (proportion of between block variation), *σ*_block_^2^ = between block variance, and *σ* ^2^ = design error variance.

## 3. Results and Discussion

### 3.1. Exploratory Data Analysis

Summary statistics for all parameters evaluated indicated that total weeds were more variable than organic matter; the percent relative standard deviations were 9.04 and 49.36 for organic matter and weeds, respectively (Table 1).* Lamium amplexicaule*, commonly known as henbit, was the most variable parameter with a relative standard deviation of 144.04%. Both* L. amplexicaule* and* Stellaria media *(chickweed) do not depend on wind for seed dispersal and tend to drop their seeds locally, perhaps contributing to patchiness. There was significant but weak correlation between organic matter and MUST (*r* = 0.30) and STEME (*r* = 0.28) (Table 2). The total number of weeds was highly correlated with BRDLF (*r* = 0.92), GRASS (0.69), and STEME (0.73). While Gaston et al. [9] found that weed densities were significantly greater in areas that had higher organic C and finer soil texture, in our study organic matter was not correlated with the total number of weed seeds. The positive correlation between weed density and both organic carbon and clay observed by Gaston et al. [9] may have been caused by an interaction with herbicides, which are more effective in lower organic matter, sandier soils.

### 3.2. Analysis of Spatial Variability and Mapping

**Figure 3:**
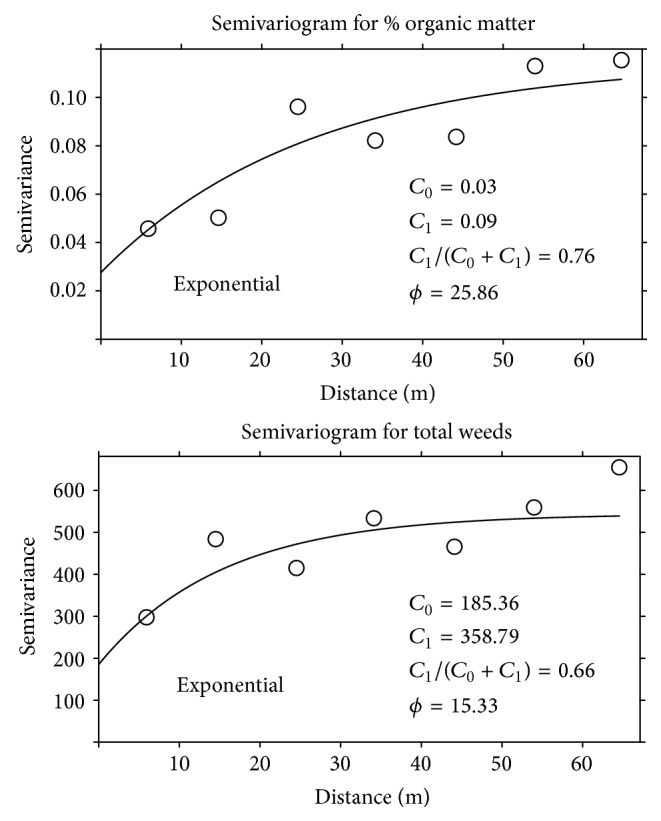
Omnidirectional semivariograms for organic matter and total weeds. *C*_0_ = nugget, *C*_1_ = sill, *C*_1_/(*C*_0_ + *C*_1_) = proportion of autocorrelation, and *ϕ* = range.

Semivariograms for both organic matter and total weeds were fit to omnidirectional exponential models. Both parameters displayed spatial structure, though there was more autocorrelation for organic matter than total weeds (*C*_1_/(*C*_0_ + *C*_1_) = 0.76 and 0.66 for organic matter and weeds, resp., Figure 3). Gaston et al. [9] also found more spatial structure in physiochemical soil properties than among weed populations. However, their minimum lag distance was 60 m compared to our 5 m minimum lag and spatial structure at a scale below the minimum lag distance will be missed. Goudy et al. [21] found that the ranges of autocorrelation for 3 different weed species varied from 11 to 62 m.

**Figure 4:**
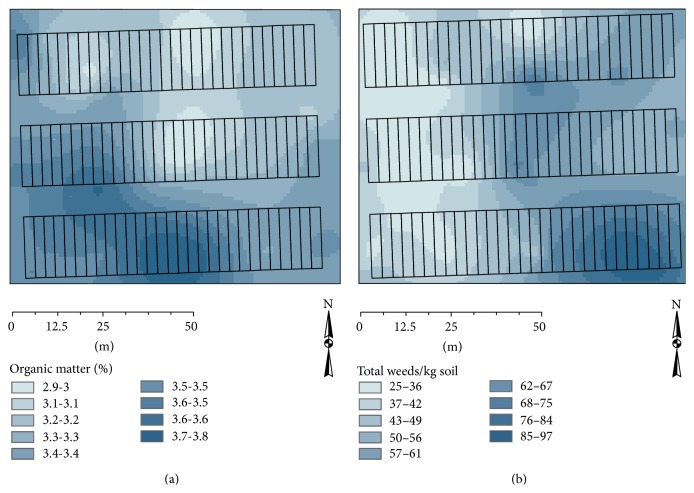
Isopleth maps for organic matter (a) and total weeds (b) were calculated using kriging with the* gstat* package in R.

Mapping revealed a trend toward increasing organic matter from the northwest of the experiment area to the southwest (Figure 4(a)). Weed populations were largest in the southeast corner (Figure 4(b)).

### 3.3. Ad Hoc Power Analysis

The completely randomized (CR) design yielded low statistical power for both organic matter and weeds (0.10 and 0.06, resp.). Since different subplots were included in each blocking pattern the CR design was run for each grouping of blocks. However, all of the CR designs produced essentially the same low statistical power for organic matter and total weeds.

**Figure 5:**
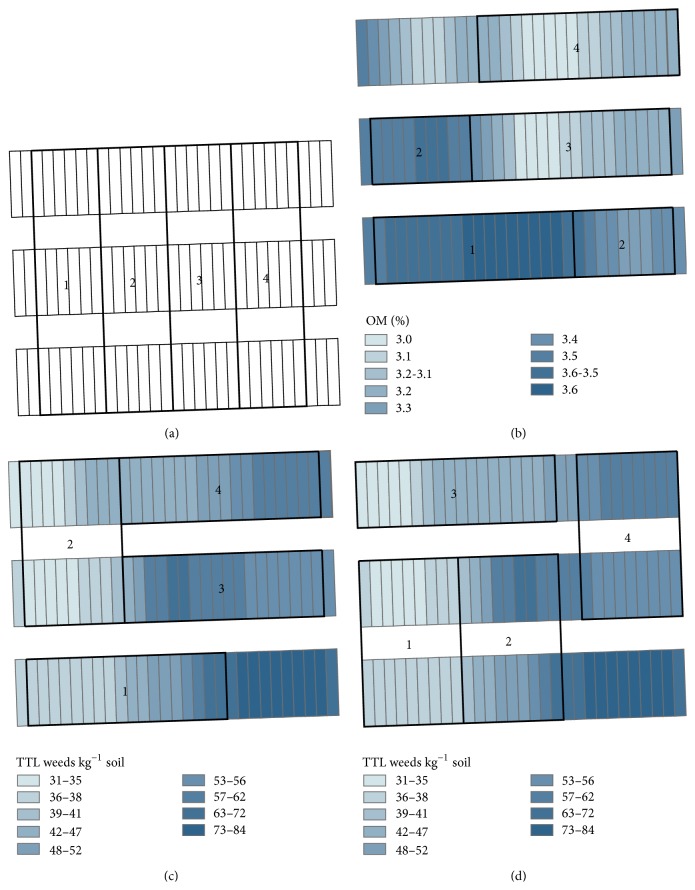
Subplot means for organic matter (b) and weeds (c-d) and four different blocking patterns evaluated for blocking efficiency with power analysis: blocking from west to east (a), blocking to optimize organic matter (b), blocking to optimize weeds, version 1 (c), and blocking to optimize weeds, version 2 (d). TTL weeds = total weeds, OM = organic matter.

The west to east blocking model (Figure 5(a)) increased both Power_OM_ and Power_weeds_ to 0.14. The organic matter model yielded the highest power, 0.98, for a response variable. This model did not affect power for weeds compared to a completely randomized design (Power_weeds_ = 0.06). Blocking to optimize weeds version 2 was the best model for improving weed power but only increased Power_weeds_ to 0.42 and increased Power_OM_ to 0.52. The model to optimize weeds version 1 increased Power_weeds_ to 0.19 and increased Power_OM_ to 0.84 (Table 3).

There was low correlation between organic matter and total weeds (Table 2). Therefore, it is not surprising that no single blocking model optimally increased Power_OM_ and Power_weeds_. We chose the weeds version 1 model as the blocking pattern for this long-term experiment. This model increased Power_OM_ to 0.84 and Power_weeds_ to 0.19. Choosing this model favored a more robust statistical model for OM than for weeds but also increases weed power compared to a completely randomized design. This model also avoided the weediest portion of the field.

## 4. Conclusions

The underlying assumption in the power analysis was that our goal was to detect a 10% difference in weeds and a 5% difference in organic matter (0.17% change in organic matter and 4.9 weeds kg^−1^). These numbers are somewhat arbitrary but provide a basis for a relative comparison of how plots can be formed into homogenous groups for blocking. The actual power of the statistical analyses could be recalculated after implementing treatments and gathering data on the plot-level, but doing the exercise a priori provides the best opportunity to reduce overall experimental error through efficient blocking.

The spatial heterogeneity of soil and weed populations poses a challenge to researchers. Unlike aboveground variability, below-ground variability is more difficult to discern without a strategic sampling pattern and a suite of tests. We demonstrated that through mapping key soil parameters we could formulate a much more efficient blocking pattern than the west to east model, which was actually the first model considered. The exercise was more successful for organic matter than for weeds, and, due to the lack of correlation between the two parameters evaluated, none of the blocking patterns examined produced high statistical power for both parameters.

Spatial analysis indicated that weeds had a shorter range of autocorrelation than organic matter and were therefore more patchy (*ϕ* = 15 m for weeds versus *ϕ* = 26 m for organic matter). This works against finding contiguous blocks large enough to accommodate the desired experimental plot size and is an argument for examining blocking patterns that are not contiguous. Blocking is also useful for managing field activities and data collection. For example, if an analysis or field operations take more than one day it is good practice to work block by block to manage variability in weather or time-dependent processes [1]. Because of this researchers are reticent to create complicated patterns, and we too strove to balance strategically placed blocks with an easy-to-follow design.

## Figures and Tables

**Table 1:** Statistical summary for soil and weed parameters in the long-term reduced tillage experiment, Puyallup, WA, 2011.

Parameter	Description	Min	Median	Mean	Max	IQR	%RSD
Organic matter	% via loss on ignition	2.6	3.3	3.3	3.9	0.4	9.0
TTL	Total weeds, g kg^−1^	12.0	45.0	48.7	117.0	37.3	49.4
STEME	*S. media*, g kg^−1^	2.0	11.0	16.1	69.0	16.8	90.1
GRASS	Grass weeds, g kg^−1^	4.0	13.5	16.2	50.0	13.8	60.6
LAMAM	*L. amplexicaule*, g kg^−1^	0.0	1.0	3.0	23.0	4.0	144.0
MUST	Mustards, g kg^−1^	0.0	5.0	8.9	41.0	10.0	116.7
OTHER	Other weeds, g kg^−1^	0.0	4.0	4.5	23.0	5.0	100.2
BRDLF	Broadleaf weeds, g kg^−1^	4.0	29.5	32.5	77.0	26.8	57.3

Min = minimum, Max = maximum, IQR = interquartile range, and %RSD = relative standard error.

**Table 2:** Coefficients of correlation between organic matter and weed data, Puyallup, WA 2011. OM = organic matter, STEME = *Stellaria media*, GRASS = total grass, LAMAM = *Lamium amplexicaule*, MUST = mustard species, BRDLF = broadleaf weeds, and TTL = total weeds.

	OM	STEME	GRASS	LAMAM	MUST	OTHER	BRDLF	TTL
OM	1.00							
STEME	0.28							
GRASS	0.07	0.34^*∗*^						
LAMAM	0.10	0.10	0.05					
MUST	0.30^*∗*^	0.06	0.12	0.17				
OTHER	0.18	0.02	0.20	0.06	0.12			
BRDLF	0.12	0.76^*∗∗*^	0.37^*∗∗*^	0.20	0.50^*∗∗*^	0.31^*∗*^		
TTL	0.12	0.73^*∗∗*^	0.69^*∗∗*^	0.16	0.44^*∗∗*^	0.32^*∗*^	0.92^*∗∗*^	1.00

^*∗*^Significant at *p* < 0.05. ^*∗∗*^Significant at *p* < 0.01.

**Table 3:** Power to detect a 5 percent difference for organic matter and a 10 percent difference for total weeds for a completely randomized design and 4 different blocking scenarios. *α* = 0.05, number of groups = 6, *n* = 4.

Model	Power_OM_	Power_weeds_
Completely randomized design	0.10	0.06
Blocking from west to east	0.14	0.14
Blocking to optimize organic matter	0.98	0.06
Blocking to optimize weeds v1	0.84	0.19
Blocking to optimize weeds v2	0.52	0.42
